# The Relationships between Process Parameters and Polymeric Nanofibers Fabricated Using a Modified Coaxial Electrospinning

**DOI:** 10.3390/nano9060843

**Published:** 2019-06-02

**Authors:** Honglei Zhou, Zhaorong Shi, Xi Wan, Hualing Fang, Deng-Guang Yu, Xiaohong Chen, Ping Liu

**Affiliations:** School of Materials Science & Engineering, University of Shanghai for Science & Technology, Shanghai 200093, China; zhouyutian007@163.com (H.Z.); 1726410207@st.usst.edu.cn (Z.S.); 1626418102@st.usst.edu.cn (X.W.); 1626410102@st.usst.edu.cn (H.F.); cxh992@163.com (X.C.)

**Keywords:** coaxial electrospinning, nanofibers, process parameter, Taylor cone, straight fluid jet, spreading angle

## Abstract

The concrete relationship between the process parameters and nanoproduct properties is an important challenge for applying nanotechnology to produce functional nanomaterials. In this study, the relationships between series of process parameters and the medicated nanofibers’ diameter were investigated. With an electrospinnable solution of hydroxypropyl methylcellulose (HPMC) and ketoprofen as the core fluid, four kinds of nanofibers were prepared with ethanol as a sheath fluid and under the variable applied voltages. Based on these nanofibers, a series of relationships between the process parameters and the nanofibers’ diameters (*D*) were disclosed, such as with the height of the Taylor cone (*H*, *D* = 125 + 363*H*), with the angle of the Taylor cone (*α*, *D* = 1576 − 19*α*), with the length of the straight fluid jet (*L*, *D* = 285 + 209*L*), and with the spreading angle of the instable region (*θ*, *D* = 2342 − 43*θ*). In vitro dissolution tests verified that the smaller the diameters, the faster ketoprofen (KET) was released from the HPMC nanofibers. These concrete process-property relationships should provide a way to achieve new knowledge about the electrostatic energy-fluid interactions, and to meanwhile improve researchers’ capability to optimize the coaxial process conditions to achieve the desired nanoproducts.

## 1. Introduction

Nanofibers, as a special kind of nanomaterial, have the unique physical properties of an individual nanoscale diameter assembled into a macroscale film, which endow them with the capability to connect the invisible nano world with the visible macro world [1,2,3,4]. Correspondingly, electrospinning, as a useful nanofabrication technique, provides a direct linkage between the macro scale spinneret and working fluids with the solid nanofibers through a typical “top-down” manner regardless of whether the working fluid numbers are treated simultaneously [5,6,7,8,9,10,11]. This process, on one hand, is a very simple and straightforward one. But on the other hand, the conversion is a very complicated process with many uncertain and changeable factors, involving several disciplines such as electrostatic dynamics, hydrodynamics, and polymeric rheology [12,13,14,15,16]. Thus, it is not strange that although numerous publications have reported the potential applications of electrospun nanofibers in a wide variety of fields, such as energy [17,18,19], environment [20,21,22,23], medicine [24,25,26,27], food engineering [28,29,30] and tissue engineering [31,32,33], no uniform theories about this process have been put forward. Often, a mathematical model that is built on a certain working fluid fails to predict another type of working fluid. Even the prediction of the nanofibers’ diameter is often far from satisfactory [34,35,36,37].

The diameter of nanofibers has a close relationship with their functional performance, which has prompted researchers to try their best to reduce their diameter [38,39]. Based on the experimental parameters, there are often three kinds of methods for realizing this goal. One involves the properties of working fluids, such as polymer concentration, viscosity, surface tension, and conductivity. The second involves the operational conditions, such as the applied voltage, the fluid flow rate, the nanofiber-collected distance, and the nozzle diameter of spinneret. And the third is manipulation of the environmental situation, such as the temperature, the humidity, the possible vacuum, and even hot air blowing [38,39,40,41,42]. These methods have achieved positive results in downsizing the nanofibers. However, few of them could give an accurate quantitative relationship between the experimental parameters and the resultant nanofibers’ diameter. This is because too many factors can exert their influences on the preparation processes simultaneously. What is more, few publications in the literature have noticed the process parameters that can be utilized to characterize the electrospinning working processes. These parameters include those of the Taylor core (its height and angle, *H* and *α*), the straight fluid jet (its length, *L*), and also the bending and whipping region (its spreading angle, *θ*) [43].

Electrospinning was revived from 1995 and was once regarded as a successive splitting process within the atomization region for creating nanofibers [44]. Almost ten years later the instable region was recorded as a high frequency bending and whipping phenomenon [45]. Then, the extremely fast drawing/drying process of working fluid during electrospinning was divided into three steps (Taylor cone, straight fluid jet and instable region). Some variables in these steps are commonly regraded as the fundamental process parameters [46,47]. These parameters, on one hand, reflect the instantaneous behaviors of working fluids under the electric field. On the other hand, they are a comprehensive reflection of the fluid-energy interactions under a series of experimental parameters. The final nanofibers are also a comprehensive result of those experimental parameters. Thus, these process parameters are essentially “precursors” of the final nanofibers’ properties, mainly their diameter, but also their smoothness, density and porosity.

Previously, a series of mathematical models have been put forward in the literature, which focus on the relationship between the experimental parameters and the final nanofibers’ quality [48,49,50]. These models have provided some knowledge on how to manipulate the working processes. However, because there are too many experimental parameters, it is very hard to simple conclude all of them in one model. Often, a slight oscillation of one experimental parameter may result in significant differences in the final nanoproducts. No matter how large the adjustment of one important experimental parameter or a very slightly normal oscillation of one insignificant experimental parameter, they should similarly exert their influences on the working fluid/the related process parameters and the resultant nanofibers/property parameters. Thus, the process parameters naturally have a higher correlation with the final products’ quality than any experimental parameters. In another word, the relationship between the process parameter and the final products can be a direct and simple tool for systematically manipulating the working process for better nanofabrication.

Coaxial electrospinning is an upgraded version of the conventional blending electrospinning process, in which a concentrate spinneret is explored to lead two different kinds of working fluids into the electric field in a core-sheath manner [51,52]. Traditionally, the sheath fluid should be electrospinnable for a successful coaxial process [53]. However, this concept was broken by modified coaxial electrospinning, in which unspinnable fluids and even organic solvent can be used as the sheath fluids for creating monolithic nanofibers with high quality [54,55].

Based on the above-mentioned knowledge, here for the first time, we investigated the relationships between the process parameters and the electrospun polymeric nanofibers’ diameter. With hydroxypropyl methylcellulose (HPMC) as a filament-forming polymeric matrix [56,57], several high voltages were applied to the working fluids. As an operational parameter, the applied voltage can both alter the process parameters and the resultant nanofibers’ size. Thus, a series of relationships between the applied voltage and the nanofibers’ sizes, between the process parameters and the nanofibers’ size were quantitatively disclosed. The process parameters could provide a more accurate result than the applied voltages for running the modified coaxial processes. In the experiments, ketoprofen (KET) was selected as the model drug, which is a nonsteroidal anti-inflammatory drug and broadly exploited to treat fever, inflammation, and pain. However, it has a very poor water solubility, which greatly limits its therapeutic effect [58,59]. The combination of KET with hydrophilic polymer in the form of electrospun nanocomposites is hypothesized to be good for the fast dissolution of KET.

## 2. Materials and Methods

### 2.1. Materials

KET was provided by Wuhan Anruike Biological Pharmaceutical Co., Ltd. (Hubei, China). HPMC powder (2910 5cps, Mn = 428,000 g/mol, methoxy content = 28.0–30.0%, hydroxypropoxy content = 7.5–12%) was obtained from Shandong Fine Chemical Co., Ltd. (Jinan, China). Ethanol and dichloromethane (DCM) were purchased from Shanghai Lingfeng Chemical Testing Co. Ltd. (Shanghai, China). All other chemicals are analytical reagents, and water was distilled twice before use.

### 2.2. Modified Coaxial Electrospraying

An electrospinnable solution consisting of 13% (w/v) HPMC and 3% (w/v) KET in a mixture of ethanol and DCM (1:1, v: v) was prepared and utilized as the core fluid. Pure solvent ethanol was used as the shell fluid. Four nanofibers referred to as F1, F2, F3, and F4 were prepared at applied voltages of 13, 14, 15, and 16 kV, respectively. For all preparations, the fiber-collected distance was fixed at 12 cm. The shell and core fluid flow rates were 0.2 and 0.8 mL/h, respectively. The modified coaxial processes were recorded using a digital camera (PowerShot A640, Tokyo, Japan).

### 2.3. Morphology of the Prepared Nanofibers

The surface morphological characterization of the prepared nanofibers was observed using scanning electron microscopy (SEM; Quanta FEG450, FEI Corporation, Hillsboro, OR, USA) at 30 kV of accelerated voltage. Before the observation, the samples were sputter-coated with gold in a vacuum. The images were analyzed by ImageJ software with over 100 different places measured.

### 2.4. Drug Fast Release Performance

20 mg of nanofibers was placed in 100 mL of phosphate buffer solution (PBS) with a pH value of 7.0. The buffer solution including samples was incubated in a shaking bath at 37 ± 0.1 °C and an agitation speed of 50 rpm. This was repeated 6 times for each kind of sample. At the predetermined time intervals, 1 mL of sample solution was withdrawn and replaced with 1 mL fresh medium. The amount of KET released from the nanofibers was measured using a UV-Vis Spectrophotometer by measuring the absorbance at 260 nm. The calibration curve was obtained, and all concentrations were evaluated in percentage as mean ± standard deviation using the following Equation (1).

(1) $$Accumulative\;release\;(\%)=\frac{Amount\;of\;drug\;release}{Amount\;of\;initial\;drug}\times 100$$

## 3. Results and Discussion

### 3.1. The Modified Coaxial Electrospinning

**Figure 1:**
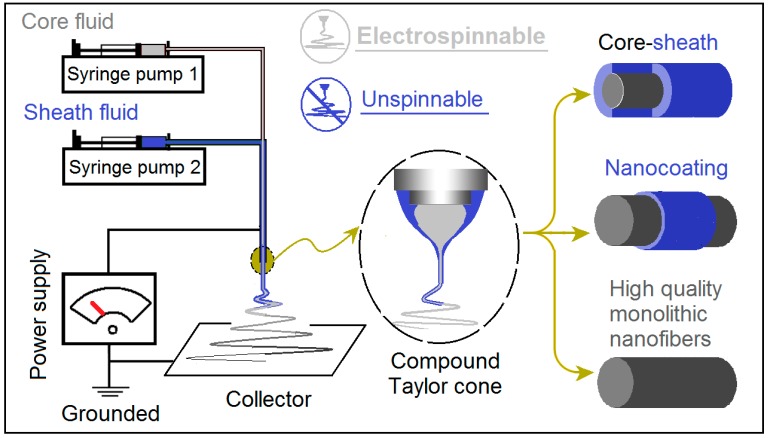
A diagram showing the modified coaxial electrospinning process, by which several different kinds of nanostructure can be created through manipulation of the unspinnable sheath fluid.

Modified coaxial electrospinning, derived from the traditional coaxial process, has several advantages over the latter. It not only can be similarly utilized to created core-shell nanostructures [60], but also can be explored to create monolithic nanofibers with high quality [43], and to favor a smooth working process with improved robustness and stability [61], just like modified coaxial electrospraying [62,63,64,65]. A diagram of modified coaxial electrospinning is shown in Figure 1.

The modified coaxial electrospinning can greatly expand the capability of electrospinning in generating new nanostructures [48,49]. This is because the electrospinnable polymers are very limited and most of them have a narrow electrospinnable window. However, the unspinnable fluids are numerous, such as all kinds of solutions, solvent, emulsions, suspensions and even slurry. The modified coaxial process makes it possible that the non-filament forming raw materials can be processed into the nanofiber format to take advantages of the huge surface areas for an improved functional performance.

**Figure 2:**
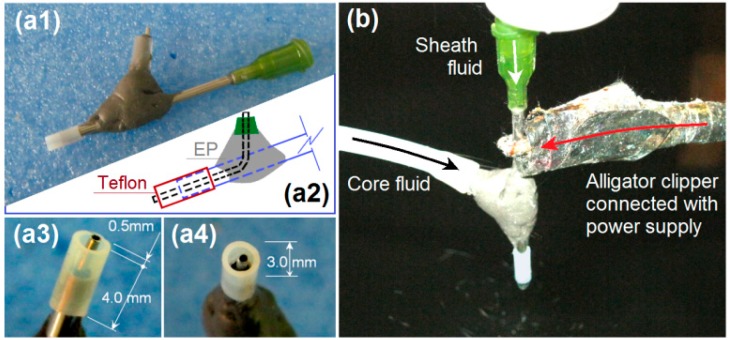
Implementation of the modified coaxial electrospinning: (**a1**), (**a3**), and (**a4**) the home-made concentration spinneret, (**a2**) a diagram showing the internal paths of sheath and core working fluids; (**b**) a digital picture of the working process.

The implementation of modified coaxial electrospinning is shown in Figure 2. The spinneret is the most important part in an electrospinning apparatus [9]. In this investigation, a special homemade concentric spinneret was developed for conducting the coaxial processes. Shown in Figure 2a1,a3,a4 are digital photos of the spinneret from different angles. The spinneret has a set of Teflon tubing as the out capillary, which leaves the inner metal capillary to project out 0.5 mm for an easy encapsulation of outer fluid on inner fluid. The internal paths of sheath and core working fluids are shown in Figure 2a2. A typical working process is exhibited in Figure 2b. An alligator clipper was utilized to convey the high voltage to the working fluid from the power supply. In this study, the sheath ethanol played an important role to keep a robust and stable working process through four routes: (1) avoiding the possible clinging of HPMC on the nozzle; (2) avoiding negative influences from the surroundings, (3) smoothing the evaporation of solvent from the core polymer solution, and (4) keeping the core fluid to be drawn for a longer time period. Thus, it is anticipated that the modified coaxial process should generate nanofibers with smaller diameter with narrower size distribution from continuous fabrication.

**Figure 3:**
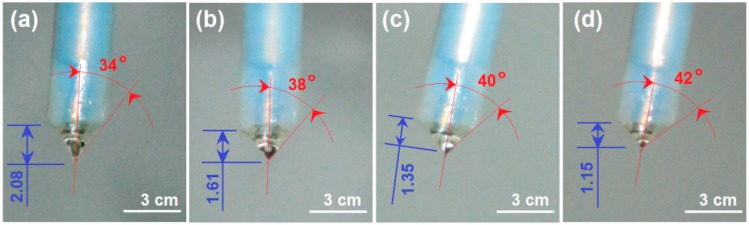
The typical changes of Taylor cone with the elevation of applied voltages (kV): (**a**) 13; (**b**) 14; (**c**) 15; (**d**) 16.

Figure 3 shows the change trends of the Taylor cone with the applied voltages. It is clear that the height of the Taylor cone (*H)* gradually decreased, whereas the angle (*α*) gradually increased as the applied voltage gradually increased. Estimated by the outer diameter of Teflon tubing of 3 cm, the height of Taylor cones were 2.08 ± 0.32, 1.61 ± 0.27, 1.35 ± 0.26 and 1.15 ± 0.24 cm and the cone angles were 34 ± 6°, 38 ± 7°, 40 ± 7° and 42 ± 6° as the voltages elevated from 13 to 14, 15, and 16 kV, respectively (*n* = 3).

**Figure 4:**
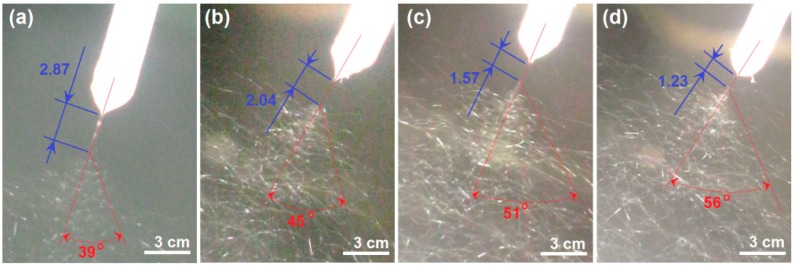
The typical changes of straight fluid jets and instable regions with the elevation of applied voltages (kV): (**a**) 13; (**b**) 14; (**c**) 15; (**d**) 16.

The alternations of the straight fluid jet and the instable region are exhibited in Figure 4. As the applied voltage increased from 13 to 16 kV, the length (*L*) straight fluid jet decreased from 2.87 ± 0.34, to 2.04 ± 0.27, 1.57 ± 0.31 and 1.23 ± 0.24 cm, whereas the spreading angle of bending and whipping increased from 39 ± 5°, to 45 ± 6°, 51 ± 5°, and 56 ± 8°, respectively (*n* = 3). Although the applied voltage has a certain electrospinnable range, within which electrospun HPMC nanofibers can be similarly created, the working fluids were highly sensitive to the changes in voltage. Moreover, because the modified coaxial electrospinning process started from the nozzle of Teflon tubing, the images of the Taylor cone, straight fluid jet and instable region could be captured simultaneously with Teflon tubing. Thus, the outer diameter could be utilized as a scale to estimate the height of Taylor cone, the cone angle, and the length of the straight jets.

### 3.2. The Electrospun KET-loaded HPMC Nanofibers and the Relationship between the Applied Voltage and the Resultant Nanofiber’s Diameter

**Figure 5:**
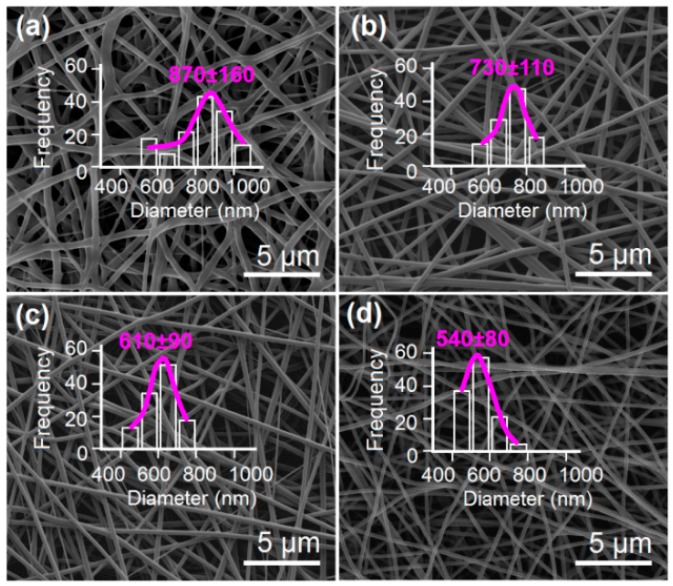
The scanning electron microscopy (SEM) images of the resultant nanofibers and their diameter distributions: (**a**) F1; (**b**) F2; (**c**) F3; (**d**) F4.

The SEM images of the prepared KET-loaded HPMC nanofibers and their diameter distributions are given in Figure 5. All the medicated nanofibers have a similarly linear morphology. No beads-on-a-string or spindles-on-a-string morphologies are found in these nanofibers, suggesting good electrospinnability of the co-dissolving solutions consisting of KET and HPMC. Nanofibers F1, F2, F3, and F4 have estimated diameters of 870 ± 160, 730 ± 110, 610 ± 90, and 540 ± 80 nm, respectively. The higher the applied voltage exerted, the smaller diameter the nanofibers had. In nanofibers F1, some clinging phenomena could be found, which should be attributed to the incomplete evaporation of solvent within the working fluids at a relatively lower applied voltage.

**Figure 6:**
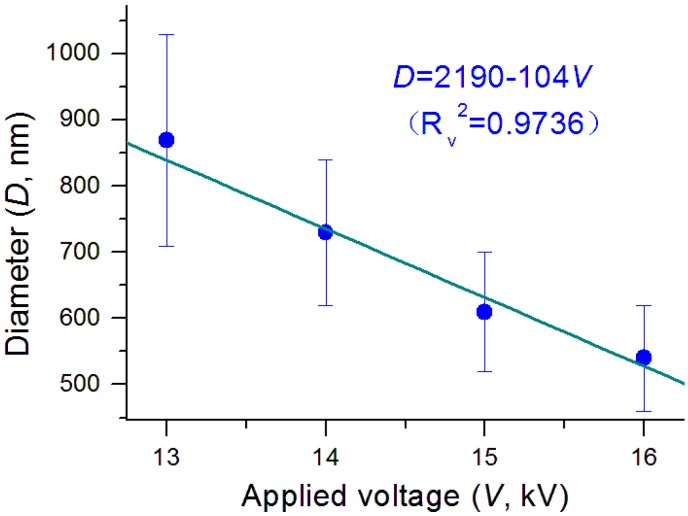
The influence of applied voltage on the size of prepared nanofibers.

Shown in Figure 6 is the influence of applied voltage on the nanofibers’ diameter. There was a clear trend that the diameter decreased as the applied voltage elevated. A linear regression suggests these two parameters have a near relationship of *D* = 2190 − 104*V*, with a correlation coefficient *R_V_*^2^ *=* 0.9736. The applied voltage is an operational parameter that can be manipulated directly by the researchers, and thus is frequently utilized to downsize the prepared nanofibers [36,37,38,39,40,41,42]. However, the effect of reducing the nanofibers’ diameter through the applied voltage is very limited in the single-fluid electrospinning process because of the formation of semi-solid substance on the surface of working fluid jets [43]. The modified coaxial electrospinning with solvent as a sheath working fluid reasonably resolves this issue. It not only prevents the pre-formation of semi-solid surface on the core fluid jets, but also keeps them to be drawn in a longer time period. Thus, modified coaxial electrospinning shows advantages over the traditional blending processes in creating high quality monolithic nanofibers, although no core-sheath nanostructures are prepared as a conventional coaxial process [66,67,68,69,70,71,72].

### 3.3. The Relationships Between the Processes Parameters and the Nanofibers’ Diameters

**Figure 7:**
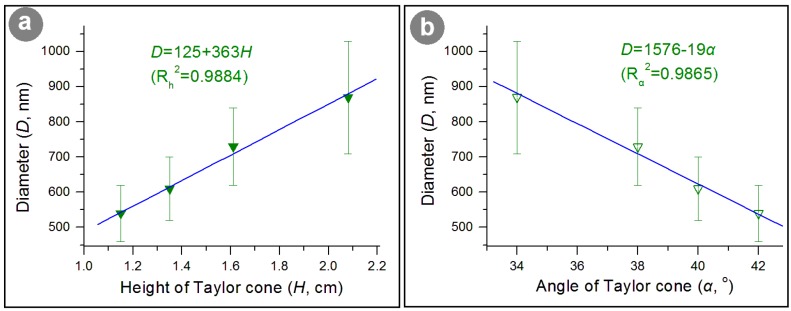
The interrelations between the nanofibers and the Taylor cone: (**a**) The relationships between the height of the Taylor cone and the nanofibers’ diameter; (**b**) The relationships between the angle of the Taylor cone and the nanofibers’ diameter.

The change in applied voltage can result in a successive response from the electropspinning working process to the final nanoproducts. Thus, the process parameters that can be utilized to characterize the working process should have a certain relationship with the nanofibers’ diameter. To fit the height and angle of the Taylor cone with the fiber’s diameter, two fine linear relationships can be found for them. Shown in Figure 7a is the change trend of diameter (*D*) with the height of the Taylor cone (*H*), whose linear equation is *D* = 125 + 363*H*, with a correlation coefficient *R_h_*^2^ *=* 0.9884. Figure 7b is the change trend of the diameter (*D*) with the angle of the Taylor cone (*α*), whose linear equation is *D* = 1576 − 19*α*, with a correlation coefficient *R_α_*^2^ *=* 0.9865. These results suggested that the applied voltage influenced both the Taylor cone’s height and angle, and the Taylor cone’s volume.

**Figure 8:**
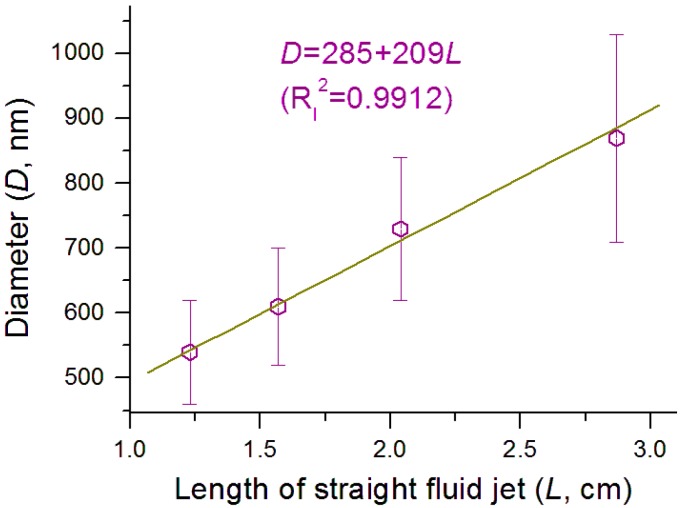
The interrelation between the length of the straight fluid jet and the fibers’ diameter.

Shown in Figure 8 is the interrelation between the nanofibers’ diameter (*D*) and the length of the straight fluid jet (*L*), whose equation is *D* = 285 + 209*L*, with a correlation coefficient *R_l_*^2^ *=* 0.9912. The higher linear correlation suggests that the longer the length of straight fluid jet, the larger diameter the nanofibers have. This suggests that the length of the straight fluid jet can be a useful process parameter for accurately predicting the diameter of the resulting nanofibers.

**Figure 9:**
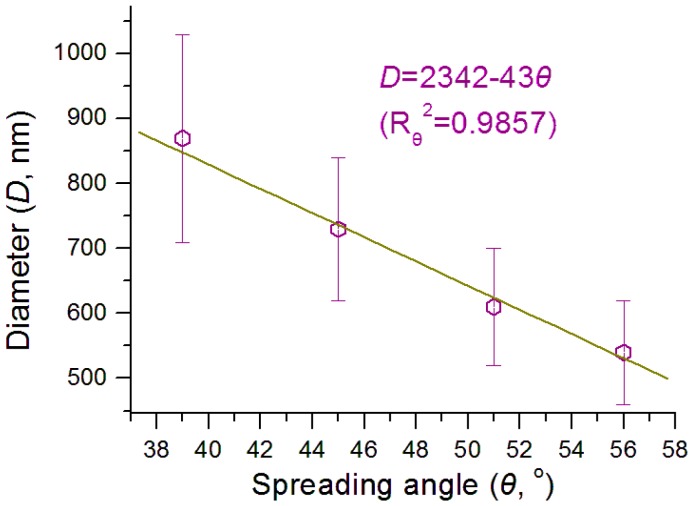
The interrelation between the spreading angle of the instable region and the fibers’ diameter.

Shown in Figure 9 is the interrelation between the nanofibers’ diameter (*D*) with the spreading angle of the instable region (*θ*), whose equation is *D* = 2342 − 43*θ*, with a correlation coefficient *R*_θ_^2^ *=* 0.9857. The larger the spreading angle is, the smaller the final nanofibers. This fine linear correlation suggests that the spreading angle of the instable region can also be a useful process parameter for accurately predicting the resultant nanofibers’ diameter.

The equations in Figure 7, Figure 8 and Figure 9 reflect the simple linear relationships between the nanofibers’ diameter and several process parameters of the Taylor cone, straight fluid jet and the instable region. These equations themselves are only applicable for the treatment of working fluids containing HPMC. They should be invalid for other polymeric solutions, however, the suggested method should be applicable to all the electrospinning processes with the typical three working steps, that is, the Taylor cone, straight fluid jet, and instable region.

In practice, any changes in the experimental conditions (including working fluid properties, operational conditions and the environment) will equally exert their influences on both the working processes and the successive solid nanofibers. Thus, the successful monitoring of the working process and accurate measurement of the process parameters would greatly increase our capability of accurate prediction about the size of final products and our capability to perform elaborate manipulation of the electrosprinning processes. As far as the measurement of process parameters is concerned, a high frequency camera can be exploited to record the working processes and to provide more accurate process parameter values than this work.

**Figure 10:**
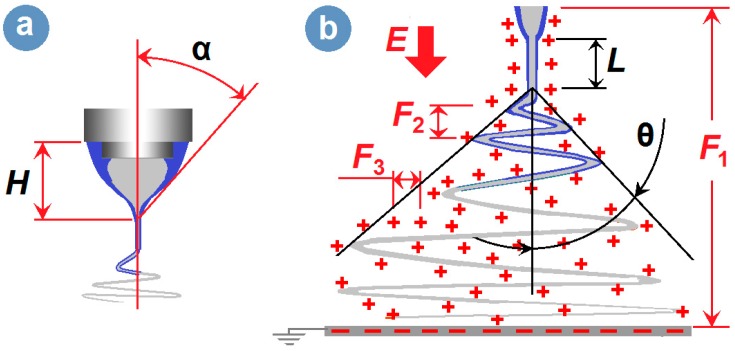
A diagram showing the typical three steps of the modified coaxial process: (**a**) The Taylor cone, (**b**) The straight fluid jet and instable region.

It is well known that the prerequisite for the initiation of electrospinning is enough electrical force resulting from a large applied voltage, which is exploited to overcome the surface tension of the droplets pumped from the nozzle of spinneret. After a critical voltage value, the elevation of applied voltage would still revise the elecrospinning process, and often the higher the applied voltage applied, the smaller the Taylor cone, the shorter the straight fluid jet, and the finer the nanofibers created. In the traditional single-fluid electrospinning, the semi-vertical angle of the Taylor cone (α), representing the sharpness of the liquid hyperboloid, is often between 32° to 46° [73]. In the modified coaxial electrospinning, the core-sheath compound Taylor-cone can still be described by its height and the Taylor cone angle (Figure 10a). Based on the literature, the angle value in the modified coaxial processes frequently goes beyond this range due to the lower elasticity and surface tensions of the unspinnable sheath working fluids [43]. Certainly, the working processes of the coaxial electrospinning are still composed of the typical three steps, that is, the Taylor cone, followed by a straight fluid jet and a bending and whipping instable region (Figure 10b).

During the electrospinning process, there are often several kinds of forces exerted on the working fluids. Shown in Figure 10b, these forces include the force (F1) between the two electrodes and the gravity (*G*, which can often be neglected), the repelling forces between the up-down bending loops (F2), and the repelling forces between the adjacent places within the fluids (F3). It is the force F3 that mainly takes charge of the size reducing effect during electrospinning. During this process, the spreading angle is a key process parameter that reflects the synergetic actions of F1, F2 and F3. The elevation of applied voltage would increase all three forces. The increase in force F3 would expand the bending and whipping loops and thus increase the spreading angle *θ,* and promote the fluid drawn effects. The increase of F2 would make the fluid move slowly for more time during the drawing process, which similarly helps to enhance the trend of enlarging the spreading angle. The F1 should accelerate the working fluids flying to the collector and reduce the spreading angle. However, the combined effect of F2 and F3 is greater than F1, and thus the apparent result is that the bigger the applied voltage, the larger value the spreading angle has.

### 3.4. The Functional Performances of the Electrospun Medicated Nanofibers

**Figure 11:**
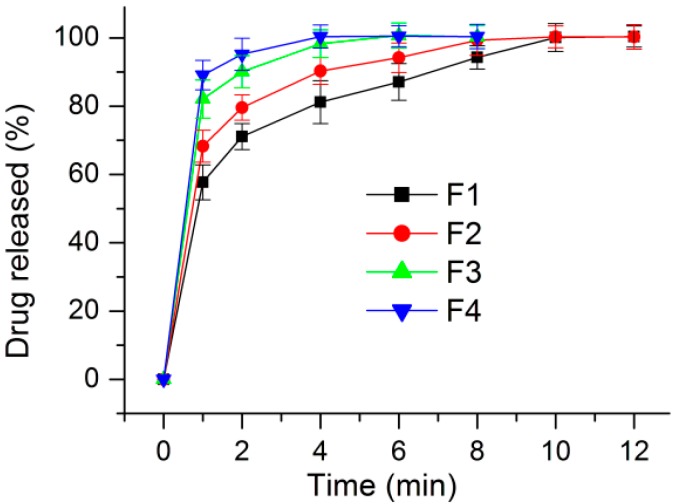
In vitro dissolution experimental results.

Shown in Figure 11 is the drug in vitro release profiles from the four types of medicated HPMC nanofibers. Although all of them were able to release over 50% of the contained drug at the first minute when they were placed into the dissolution media (57.6 ± 4.9%, 68.5 ± 4.4%, 81.8 ± 5.2%, and 89.3 ± 4.5% for nanofibers F1, F2, F3, and F4, respectively), a trend formed by them is clear. The smaller the diameter of the nanofibers, the faster the loaded drug was exhausted from the nanofibers. This can be anticipated because the smaller the diameters, the larger the surface area and the bigger the porosity; these positive factors would promote an easy dissolution of the HPMC and the loaded KET.

**Figure 12:**
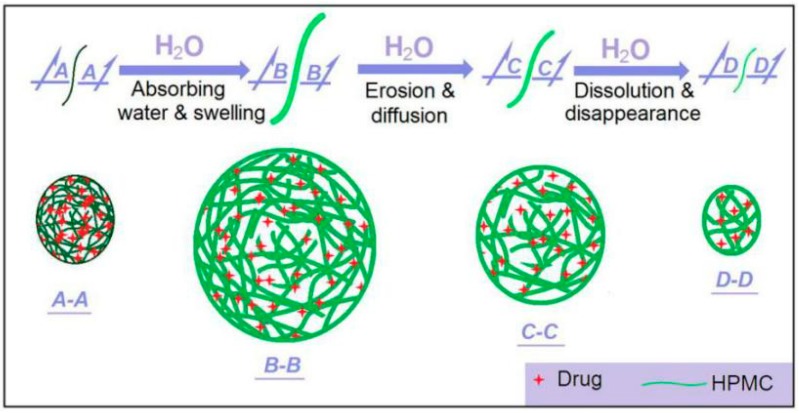
A diagram showing the drug dissolution mechanisms from the medicated ketoprofen (KET)-loaded nanofibers.

HPMC is a soluble and hydrophilic polymer. Shown in Figure 12 is a diagram about the drug release processes from the medicated nanofibers. Within the nanofibers, the drug molecules are homogeneously distributed all over them due to the extremely fast drying process of electrospinning. When these medicated nanofibers are placed into water, they will absorb water to swell gradually (from *A-A* to *B-B*). In this process the water molecules penetrate the solid nanofibers. The solid nanofibers gradually become a transparent hydrogel. Meanwhile, the drug molecules should leave the polymer chains and go into the penetrated water, and further diffuse outward to the bulk solution due to the concentration gradient. This is a drug diffusion mechanism. As the swelling goes forward, the outer layer HMPC molecules will free themselves into the bulk solution, together with the contained and penetrated drug molecules, until complete disappearance (from *B-B* to *C-C* and *D-D*). Thus, the erosion mechanism also happens here.

As the nanofiber diameters decrease, the penetration distance of water and diffusion distance of drug molecules should all decrease correspondingly. This is to say the decrease of diameter will shorten the drug release time period due to the diffusion mechanism. This should be the reasons that the smaller the nanofibers’ diameter, the more positive the results for the fast dissolution of the poorly water-soluble drug. The poor water-solubility of drugs is one of the most difficult and long-existing issues in pharmaceutics [73,74,75,76,77]. Nanotechnologies have brought new lights on resolving this problem. However, taking advantage of these advanced techniques comprises a challenge to the researchers. The present study shows a fine example to build a clear process-property-performance relationship for exploring modified coaxial electrospinning to create functional nanofibers.

## 4. Conclusions

In this study, modified coaxial electrospinning was successfully carried out using a Teflon-coated concentric spinneret. Four types of KET-loaded HPMC nanofibers were created under a variable experimental parameter—applied voltage. Based on the SEM images, the nanofibers’ diameter (*D*) was estimated and a series of relationships between it and the process parameters were disclosed, such as with the height of the Taylor cone (*H*, *D* = 125 + 363*H,* *R_h_*^2^ *=* 0.9884*)*, with the angle of the Taylor cone (*α*, *D* = 1576 − 19*α,* *R_α_*^2^ *=* 0.9865), with the length of the straight fluid jet (*L*, *D* = 285 + 209*L,* R*_l_*^2^ *=* 0.9912), and also with the spreading angle of the instable region (*θ*, *D* = 2342 − 43*θ,* *R*_θ_^2^ *=* 0.9857). All these relationships show a slightly better linear relationship than the applied voltage experimental parameter (*V*, *D* = 2190 − 104*V*, *R_V_*^2^ *=* 0.9736), as indicated by their correlation coefficients. In vitro dissolution tests verified that the smaller the diameters, the faster the release of KET from the HPMC nanofibers. These clear process-property relationships should provide a way to achieve new knowledge about the electrostatic energy-fluid interaction process, and meanwhile improve the capability of researchers in optimizing coaxial process conditions to achieve the desired nanoproducts.
